# Comparison Between Vancomycin and Gentamicin for Intraoperative Presoaking of Hamstring Graft in Primary Anterior Cruciate Ligament Reconstruction

**DOI:** 10.7759/cureus.22550

**Published:** 2022-02-23

**Authors:** Deepak Bansal, Kavin Khatri, Anshul Dahuja, Amit Lakhani, Neeraj Malhotra

**Affiliations:** 1 Orthopaedics and Traumatology, AIMC Bassi Hospital and Ludhiana Mediways Hospital, Ludhiana, IND; 2 Orthopaedics, All India Institute of Medical Sciences, Bathinda, Bathinda, IND; 3 Orthopaedics, Guru Gobind Singh Medical College, Faridkot, Faridkot, IND; 4 Orthopaedics, Dr. B.R. Ambedkar State Institute of Medical Sciences, Mohali, IND; 5 Orthopaedics, Government Medical College, Amritsar, Amritsar, IND

**Keywords:** vancomycin, gentamicin, septic arthritis, infection, anterior cruciate ligament reconstruction

## Abstract

Introduction

Postoperative infection is an uncommon complication with grave consequences following anterior cruciate ligament reconstruction (ACLR). Presoaking of the hamstring graft with antibiotics results in a lower rate of infection. The purpose of the current study was to compare the efficacy of two commonly used antibiotics, vancomycin and gentamicin, in reducing infection rates following anterior cruciate ligament reconstruction.

Methods

The retrospective study included a total of 578 patients who underwent arthroscopic anterior cruciate ligament reconstruction between June 2015 and October 2021. The timeline was categorized as the period between June 2015 to October 2018 (Vancomycin presoaking of hamstring graft) and November 2018 to October 2021 (Gentamicin presoaking of hamstring graft). All patients were examined for the development of infection, causative organism, and treatment received. Patients with intravenous drug abuse, alcoholism, steroid use, revision cases, and a prior history of infection in the knee were excluded from the study. Fisher’s exact test was used for comparison of categorical data, and Poisson regression analysis was carried out to calculate incidence rate ratios after adjusting for confounding variables.

Results

Presoaking of hamstring grafts with vancomycin was carried out in 224 patients, and gentamicin was used in 354 patients. In total, three patients in the vancomycin and four patients in the gentamicin groups developed an intraarticular infection, and the difference in infection rate between the two groups was not statistically significant (p=0.919). Coagulase-negative *Staphylococcus aureus* was isolated in four cases, Enterobacter cloacae in one, and no organism was seen in two cases. The groups were comparable in terms of age (p=0.563), smoking (p=0.84), sex (p=0.359), and operative time (p=0.09).

Conclusion

Presoaking of hamstring autografts with gentamicin intraoperatively is a good alternative to vancomycin in the prevention of infection following arthroscopic anterior cruciate ligament reconstruction.

## Introduction

There has been a marked increase in the number of cases of anterior cruciate ligament reconstruction (ACLR) worldwide. ACLR helps in the restoration of knee instability. But infection following ACLR is a devastating complication [1]. The reported incidence varies from 0.14% to 0.17% [2,3]. It may require multiple reoperations in the form of irrigation and debridement, prolonged use of antibiotics, removal of the graft, and sometimes graft revision surgery. It also has a negative impact on functional outcomes, increases the risk of early osteoarthritis, and causes graft failure [4,5].

There are a number of contributing factors that can lead to infection, like the presence of comorbid conditions like diabetes or concomitant open surgical procedures. Use of larger incisions, longer tourniquet time, and use of drain are other contributing factors [6]. Some studies have reported increased chances of infection with hamstring autografts in comparison to patellar tendon autografts and drain application [7,8]. Preoperative intravenous antibiotics given prior to skin incision may not be sufficient enough to reach minimum inhibitory concentration levels due to poor vascularity of hamstring tendons. The harvested graft is prone to infection from skin flora, and an adequate concentration of antibiotics to inhibit bacterial growth can be achieved with the local application of antibiotics over the graft [9]. The most common pathogen after ACLR is staphylococci, accounting for 90% of cases of septic arthritis [10,11]. Other bacteria like Propionibacterium and Enterobacter species are among the other bacteria isolated in cases of infection post-ACLR [12].

Prior studies had supported the use of presoaking harvested grafts in vancomycin solution to reduce the incidence of septic arthritis following ACLR [9-13]. Vancomycin is commonly used because of its properties like heat stability, safety for local use, and bactericidal action against organisms like *Staphylococcus aureus* [14]. Previous studies have shown good midterm functional outcomes with vancomycin solutions [15]. But concerns like antibiotic resistance, high cost, and graft toxicity are associated with the use of vancomycin [16]. In the recent past, studies have evaluated the effect of gentamicin solution on ACLR [9]. Gentamicin offers an advantage in terms of activity against staphylococci, Gram-negative pathogens, and pseudomonas, along with being cost-effective [9]. However, to the best of our knowledge, there is no study comparing the efficacy of vancomycin with gentamicin solution in the presoaking of hamstring autografts during ACLR.

The objective of the present study was to compare the infection rate with pre-soaking of harvested hamstring grafts in vancomycin or gentamicin during ACLR. The hypothesis of the study was that pre-soaking of grafts with either gentamicin or vancomycin would result in a similar infection rate.

## Materials and methods

A retrospective study of all the patients who had undergone primary ACLR from June 2015 to October 2021 at a large multispecialty hospital with a dedicated arthroscopy center was conducted. The study was approved by the hospital ethics committee of Ludhiana Mediways hospital, bearing the number LM/AS-2/2021. The surgeons (DB, DG, and AL) had used vancomycin for presoaking hamstring grafts prior to October 2018 and switched to the use of gentamicin in place of vancomycin subsequently. They had switched to gentamicin with literature coming up in support of gentamicin irrigation solution during ACLR to prevent infection [9,10]. 

Patients were divided into two consecutive periods: January 2015 to October 2018 (Vancomycin protocol group, group A) and November 2018 to October 2021 (Gentamicin protocol group, group B). The cases that were followed up for a period of 90 days were included in the study as considered appropriate in a previous study by Baron et al. [13] for the detection of infection after ACLR. Patients with the concomitant open procedure on an ipsilateral limb, use of non-hamstring graft, revision ACLR, history of previous infection in the same knee, intravenous drug abuse, alcoholism, and steroid use were excluded from the study. The charts of the patients were reviewed to identify the demographics of the patients, which included age, body mass index, sex, smoker or non-smoker status, and presence of diabetes. A patient was termed a nonsmoker if he had stopped smoking at least one year prior to the operative procedure. Operative parameters and procedures like operative time, meniscal repair reconstruction, and additional ligament reconstruction were also noted. All the patients were administered intravenous cefuroxime 1.5 g after sensitivity testing an hour prior to skin incision and tourniquet application. The intravenous injection was repeated in case the surgery was prolonged for more than two hours. Three patients had sensitivity to cefuroxime, and in that case, clindamycin (600 mg) intravenous was administered. The ACLR was carried out with the same protocol for preparation, draping, and surgical technique by all the surgeons.

Anterior cruciate ligament reconstruction protocol

An ACLR was performed after a diagnostic arthroscopic examination of the knee joint. The hamstring (Gracilis and Semitendinosus) grafts were harvested under tourniquet control through the anteromedial aspect of the tibia. The quadrupled graft was fixed with an endobutton (Smith and Nephew, Andover, MA, USA) on the femoral cortex and a bioabsorbable screw (Smith and Nephew) on the tibial side. In both groups, postoperatively, a total of three doses of injection cefuroxime of 1.5 g were given intravenously every eight hours. No drains were used in either group.

Protocol for presoaking hamstring autografts

The surgeons consistently followed a standard protocol in harvesting and presoaking the hamstring autografts. In vancomycin group A, hamstring tendon autografts were soaked in a 500 ml solution of 1 mg/ml concentration of vancomycin for 10 minutes [13]. They were kept in vancomycin-soaked sponges till the passage of grafts through tunnels. In gentamicin group B, 40 mg of gentamicin was added to 500 ml of normal saline in order to achieve a concentration of 80 mg/L, which is above the minimum effective concentration of 50 mg/L [9]. The grafts were soaked in the gentamicin solution and kept in presoaked sponges for the duration in a manner similar to the vancomycin group.

Follow up

Patients were followed up at one, two, six, and twelve weeks after arthroscopic ACL reconstruction by the nursing staff, and the required details were noted down. No one from the research team except the operating surgeon (DB) observed the patients in the follow-up period. The patients underwent similar post-operative rehabilitation programs with a few deviations as per the individual requirements under the supervision of a trained physiotherapist. All the patients were followed up for six months.

Detection of infected cases

Infected ACL reconstruction was defined as a requirement for an operative procedure within 90 days after the index operative procedure as described by Baron et al. [13]. Any joint effusion, increase in pain, limitation of range of motion, or fever were noted. In these patients, total leucocyte count (TLC), differential leucocyte count (DLC), C-reactive protein (CRP), and erythrocyte sedimentation rate (ESR) were checked and subjected to knee aspiration. In the case of no aspirate, patients were subjected to a biopsy of the involved knee. Knee aspirates were sent for TLC, cultures, and antibiotic sensitivity analysis.

Statistical analysis

The statistical analysis of the current study was performed with descriptive and inferential statistical analyses. Categorical variables were assessed by the chi-square test and Fischer’s exact test. Incidence rate ratios (IRR) with a 95% confidence interval were calculated using Poisson regression analysis. At one point of time, only two variables were examined as there was a concern about overfitting the multivariate model with a low infection rate after ACLR. Multiple models were created while controlling for a single variable (operative time, associated ligamentous procedures, and body mass index). Multivariate and univariate analyses were performed using SPSS 16.0 software (IBM Corp., Armonk, NY), and a p-value of <0.05 was considered statistically significant.

## Results

A total of 712 patients underwent primary ACLR using hamstring grafts between June 2015 and February 2021. However, 134 patients were lost to follow-up. Among the 578 patients, group A (the vancomycin protocol) consisted of 224 patients, and group B (the Gentamicin protocol) consisted of 354 patients. 90 patients (15.57%) had diabetes mellitus and 138 patients (23.87%) were active smokers. There was no significant difference between the two groups (Table 1) in terms of age (p=0.563), sex (p=0.685), smoking (p=0.084), the prevalence of diabetes (p=0.254), and operative time (p=0.092).

**Table 1:** Demographic characteristics of the patients with the use of vancomycin (group A) or gentamicin (group B) for presoaking of Hamstring autografts. *Depicted as mean and standard deviation. ^#^Denotes the number of patients.

	Vancomycin protocol group (N=224)	Gentamicin protocol group (N=354)	P-value
Age^*^	28.6±10.9	28.1±9.6	0.563
Male^#^	156	259	0.359
Body mass index^*^	22.65±4.7	22.13±5.1	0.218
Smoking^#^	67	83	0.084
Diabetes^#^	17	23	0.254
Operative time^*^	128.78±22.1	124.72±31.5	0.092

Intraarticular infection

**Figure 1:**
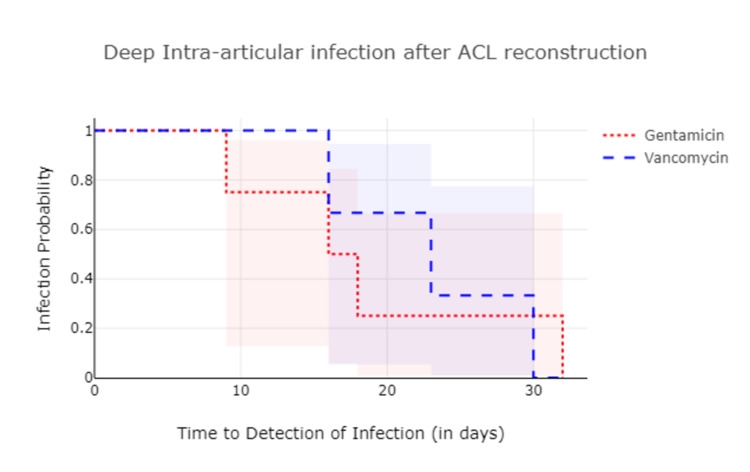
Kaplan-Meier curve showing time for development of deep intra articular infection with vancomycin and gentamicin following anterior cruciate ligament reconstruction.

In total, seven cases (1.2%) had developed an infection after ACLR, and among them, three cases were in the vancomycin group and four cases in the gentamicin group (Tables 2-4). The difference in infection rate was not statistically significant between the two groups (p=0.909). We found no association between the operating surgeon and infection rate (Table 5). The average duration following ACL reconstruction and the appearance of infection was 12.3±14.1 days in group A and 13.2±13.9 in group B (Figure 1). In multivariate analysis, the cofounding factors like body mass index, operative time, and other ligamentous procedures were controlled and checked for infection rate with gentamicin in comparison to vancomycin. It was determined that there was no significant difference in the infection rate between the two drugs (Table 6).

**Table 2:** Patient demographics and intraoperative variables of patients with infection following ACLR. *Depicted as mean and standard deviation. ^#^Denotes the number of patients with percentage in parentheses. The data were not normally distributed.

Parameter	Vancomycin protocol group [N=3; 1.34%]	Gentamicin protocol group [N=4; 1.13%]	IRR (95% CI)	P-value
Age (in years)^*^	28.6±10.4	31.4±9.8	−2.8 (−22.520 to 16.920)	0.730
Male^#^	2 (66.67%)	4 (100%)	0.1852 (0.0053 to 6.4761)	0.352
Body mass index (BMI)^*^	21.4±5.4	23.6±4.8	−2.2 (−12.112 to 7.712)	0.570
Diabetes^#^	0 (0%)	1 (25%)	0.4286 (0.0130 to 14.0823)	0.634
Smoking^#^	0	2 (50%)	0.2571 (0.0091 to 7.2732)	0.425
Operative time (minutes)^*^	120±24.1	122.1±23.4	−2.1 (−48.596 to 44.396)	0.116
Meniscectomy^#^	1 (33%)	0 (0%)	3.8571 (0.1174 to 126.7403)	0.448
Meniscal repair^#^	0 (0%)	1 (25%)	0.4286 (0.0130 to 14.0823)	0.6344
Other ligamentous procedure^#^	1 (33%)	1 (25%)	1.3333 (0.0571 to 31.1228)	0.8579

**Table 3:** Patient characteristics of Infected cases in vancomycin group.

Case	Age (years)	Sex	Days from ACLR to diagnosis of infection	Blood parameters	Joint fluid aspirate	Culture/positive tissue biopsy	Hospital stay (days)
1	32	Male	23	TLC:13,100 PMN: 85% ESR: 34 CRP: 24	Turbid Yellow TLC: 78100 PMN: 96%	Enterobacter cloacae	34
2	27	Male	30	TLC:11,300 PMN: 82% ESR: 40 CRP: 18	Cloudy Yellow TLC: 85000 PMN: 91%	Coagulase negative *S. aureus*	27
3	31	Male	16	TLC:11,600 PMN: 80% ESR: 45 CRP: 21	Turbid Yellow TLC: 88,700 PMN: 97%	Coagulase negative *S. aureus*	31

**Table 4:** Patient characteristics of infected cases in gentamicin group.

Case	Age (years)	Sex	Days from ACLR to diagnosis of infection	Blood parameters	Joint fluid aspirate	Culture/positive tissue biopsy	Hospital stay (days)
1	23	Male	18	TLC:11,300 PMN: 88% ESR: 43 CRP: 13	Turbid Yellow TLC: 54600 PMN: 80%	Coagulase negative *S. aureus*	24
2	27	Male	9	TLC:14,400 PMN: %83 ESR: 45 CRP: 25	Turbid Yellow TLC: 80000 PMN: 89%	Coagulase negative *S. aureus*	42
3	21	Male	16	TLC:12,400 PMN: 78% ESR: 36 CRP: 15	Turbid Yellow TLC: 73,300 PMN: 83%	No growth	28
4	30	Female	32	TLC:11,500 PMN: 82% ESR: 34 CRP: 26	Turbid Yellow TLC: 79,100 PMN: 83%	No growth	24

**Table 5:** Infected cases reported by the individual operating surgeons.

	Total operations	Infected cases	Infection rate (%)
Surgeon A	267	3	1.12
Surgeon B	197	3	1.52
Surgeon C	114	1	0.8

**Table 6:** Multivariate analysis to assess the association of gentamicin presoaked hamstring graft with infection. The effect of gentamicin on decreasing infection was ascertained by creation of multiple models with controlling for a single variable. There was no significant difference in infection rate between presoaking of either gentamicin or vancomycin.

Controlled variable	Incidence rate ratio (95% confidence interval)	P-value
Body mass index	1.355 (0.432 to 3.942)	0.541
Operative time	0.417 (0.115 to 1.967)	0.154
Other ligamentous procedure	2.004 (0.252 to 13.643)	0.581

Superficial infection

Superficial infection was reported in eight cases. The difference between the vancomycin protocol group (5/224) versus the gentamicin group (3/354) was not statistically significant (p=0.14). The superficial infection was subsided with regular dressings and the use of oral antibiotics for two weeks.

Other complications

There were 15 cases of graft failure due to non-infective reasons in the gentamicin group, compared to 10 cases in the vancomycin protocol group (p=0.896). Three of the failed cases in the gentamicin group were professional Kabaddi players (a contact sport popular in rural India) who were injured while playing. One patient had a fall from a motorcycle and experienced ACL loosening along with a fractured shaft of the femur. Eight cases had aseptic loosening of the graft (improper tunnel positioning) and three cases had missed lateral collateral injuries leading to subsequent failure of the graft.

In the vancomycin group, six patients had aseptic loosening of hamstring grafts due to improper tunnel positioning, one patient had a missed lateral collateral injury, and three patients had road traffic accidents.

One case in vancomycin and two cases in the gentamicin group experienced stiffness and required manipulation under anesthesia. In seven cases, deep vein thrombosis (DVT) was recorded, while pulmonary embolism was not reported in any case in the study. There were more cases of DVT recorded in the gentamicin group (n=3) as compared to the vancomycin group (n = 4), but the difference was not statistically significant (p=0.567).

## Discussion

In the current retrospective study of 578 ACL reconstructions, there was no statistically significant difference in the intra-articular infection rate with presoaking of hamstring grafts intraoperatively with either vancomycin or gentamicin solution. The most common organism isolated for intra-articular infection was coagulase-negative *S. aureus*. In these cases, the patients were treated with arthroscopic irrigation and intravenous antibiotics.

Earlier studies had identified smoking and diabetes as risk factors for deep intra-articular infection after ACL reconstruction [7,17]. Cancienne et al. [18] reported tobacco use as an independent risk factor for infection after ACLR in a cohort of 13,358 patients. In their study, Brophy et al. [7] detected diabetes as an independent risk factor for infection. However, the present study could not substantiate the same. This may be due to the fact that the current study may not be adequately powered to evaluate the association between smoking and diabetes with infection. The diabetic patients in the study had shown acceptable glycemic control with Hb1Ac of 7.2±1.4%.

Preito et al. [19] in their study on the identification of points of graft contamination concluded that the maximum chance of graft contamination is during graft preparation followed by graft harvesting. So, the rationale for presoaking the graft came up and the "vancomycin wrap" technique was subsequently described by Vertullo et al. [20]. Regarding the preference of antibiotics to be used for presoaking grafts, it should cover the organism predictably causing infection. In addition, it should have minimal side effects, be cheaper, and have fewer chances of antimicrobial resistance.

While gentamicin lavage has been extensively used in open fracture and total joint arthroplasty, there are fewer instances of its use in ACLR. Gentamicin is cheaper and has a broader spectrum in comparison to vancomycin. Its mechanism of action is by binding to the 30s subunit of the bacterial ribosome and inhibiting multiplication. The drug acts in a short span of time to achieve the required levels of bacterial inhibition, which is desired intraoperatively. It is effective against both Gram-positive and Gram-negative bacteria [21,22]. Gentamicin has been proven to be effective in open fractures contaminated with *S. aureus*. Post ACLR, *S. aureus* is the most common organism isolated from cases of septic arthritis [23]. Gentamicin has low chondrotoxicity and is, therefore, a safer drug to use in joints [24]. There are reports of hypersensitivity reactions in up to 15% of the patient population with vancomycin, whereas with gentamicin the numbers are less than 2% [25]. There are incidences of contact dermatitis with topical application of aminoglycosides, but no case of the same was reported in the current study as there was an abundant saline wash of the skin after the procedure. Bortnem et al. [26] studied the efficacy of gentamicin lavage in 100 cases of joint replacement and concluded that it is cost-effective to use gentamicin in joint irrigation. Yazdi et al. [9] in their retrospective study cohort of 1464 ACL cases reported an incidence of infection of 0.23% of cases with the use of gentamicin as an irrigation solution.

In many in vitro studies [24], vancomycin demonstrated low chondrotoxicity in comparison to other antibiotics. Grayson et al. [27] demonstrated absorption of vancomycin by bovine tendons and subsequently released the same over a 24-hour period. Schüttler et al. [28] in their study demonstrated the safety profile of vancomycin. There was no evidence of any change in the biomechanical properties of tendons with the use of vancomycin. Vancomycin acts against Gram-positive organisms. In their retrospective analysis, Vertullo et al. [20] reported a decrease in infection rates from 1.4% to 0% in 870 cases with the use of vancomycin solution for soaking hamstring grafts. Another retrospective study of 1640 cases of ACL reconstruction by Baron et al. [13] reported a decrease in the incidence of infection from 1.2% to 0.1% with the use of vancomycin solution. Offerhaus et al. [16] in their study of 1779 cases reported a 0% deep postoperative infection rate at the end of 28 months with soaking of the hamstring in vancomycin solution as compared to systemic antibiotic prophylaxis alone.

There is a risk of resistance with the routine use of systemic antibiotics. Studies in cardiovascular procedures and spine surgery have not reported any increased resistance risk with the intrawound application of antibiotics. The literature is limited regarding the risk of resistance associated with the intra-articular use of antibiotics. Ghobrial et al. [29] in their study on spinal fusions reported polymicrobial growth with local powdered application of vancomycin. In their study on prosthetic joint replacement, Hansen et al. [30] did not report the emergence of antimicrobial resistance with the use of antibiotic-laden cement. In our study, gentamicin resistance was not reported in any of the organisms isolated from intraarticular infection.

Limitations

The current study had some limitations. First, it was a retrospective study with no randomization. Second, some cases after developing infection may not have reported back for treatment. Third, due to the inclusion of the gentamicin group in the later period of the study, might have led to performance and chronological bias as improved surgical technique and better postoperative care could have reduced the risk of infection. Fourth, the follow-up period of 90 days for the detection of infection could have introduced detection bias as some cases might have presented late at other institutions. Fifth, a lower number of infected ACLRs limited the power of the study. However, we know that infection is a rare complication after ACLR, so the lower number is expected even with a large sample size.

## Conclusions

The soaking of hamstring grafts in either vancomycin or gentamicin is associated with a similar incidence of postoperative infection following ACLR. Both antibiotics are effective against coagulase-negative *S. aureus*, which is among the most commonly isolated organisms following intraarticular infection after ACLR. They also demonstrated low chondrotoxicity in comparison to other antibiotics. Among the two antibiotics, gentamicin is cheaper in comparison to vancomycin. So, gentamicin is a safe, cheap, and effective alternative to vancomycin for presoaking autografts and prevents a rare but devastating complication of intraarticular infection.
